# Where is the family voice? Examining the relational dimensions of the family- healthcare professional and its perceived impact on patient care outcomes in mental health and addictions

**DOI:** 10.1371/journal.pone.0215071

**Published:** 2019-04-12

**Authors:** Sophie Soklaridis, Miriam McCann, Jacquelyn Waller-Vintar, Andrew Johnson, David Wiljer

**Affiliations:** 1 Centre for Addiction and Mental Health, Toronto, Ontario, Canada; 2 Department of Psychiatry, Faculty of Medicine, University of Toronto, Toronto, Ontario, Canada; 3 Department of Family and Community Medicine, Faculty of Medicine, University of Toronto, Toronto, Ontario, Canada; 4 The Wilson Centre for Research in Education, University Health Network and University of Toronto, Toronto, Ontario, Canada; 5 University Health Network, Toronto, Ontario, Canada; 6 Institute of Health Policy, Management and Evaluation, University of Toronto, Toronto, Ontario, Canada; Institute of Mental Health, SINGAPORE

## Abstract

**Background:**

We explored the relationship between family members and healthcare professionals (HCPs), specifically how family members can influence the course and outcome of patient care for youth. Exploring this under-researched area provided an opportunity to understand the tripartite relationship between the family, the youth experiencing mental health problems or substance use concerns and their HCP.

**Methods:**

A qualitative research design was used to gain a full understanding of how family members experience relationships with HCPs. We interviewed 21 family members using semi-structure questions to explore the type of relationships formed between HCPs and family members throughout a patient’s course of care, the family member’s perceived role in the care of their youth accessing mental health or addiction services and the family member’s awareness of formalized structures (i.e., hospital rules, policies) and resources that support family involvement.

**Results:**

Within a relationship-centred framework, four themes, with various sub-themes emerged from the interviews: 1) The family member–HCP relationship regarding creating a positive impression, being an extension of the patient and the discovery of “pink flags”; 2) The family member–youth–HCP relationship regarding the receptivity of youth to family involvement and a youth’s individual right to privacy; 3)The family member’s relationship to self with regard to the situation being a family illness; and 4) The family member’s relationship with friends, family and peers regarding the feelings of loneliness, stigma and shame and the lack of understanding about mental health problems and substance use.

**Conclusions:**

Our study provided in-depth information about the importance of family involvement in the care and health outcomes of youth who are accessing mental health and addiction services. Family members experienced and conceptualized their relationships with HCPs, their youth, themselves and their friends and peers as active interactions that influenced the course and outcomes of care. Future studies are needed to collect the multiple perspectives of youth and HCPs alongside with the family perspectives.

## Background

It is well known that family members are particularly important in the care and treatment of youth who experience mental illness or substance use problems [1]. Families often have an in-depth knowledge of the youth receiving care and are a consistent support system for youth, helping them manage their illness day-to-day and through a crisis [1, 2]. Family members are often the first to recognize signs of relapse and are thus able to connect youth to services and healthcare professionals (HCPs) [3]. Although there is increased evidence to support the benefits of incorporating the family as a resource and partner in mental healthcare and addictions, this concept is often not translated into clinical practice [4, 5]. For example, in a systematic review on implementing family involvement in the treatment of patients with psychosis, the authors found 43 studies in total. Of these studies, 32 focused solely on staff perspectives [3]. While other research focused on the potential role of the family and their perceived psychosocial support needs [6,], none included information on how the relationship between the HCP and family can influence the course and outcome of patient care. Barriers to creating those partnerships included privacy concerns, fears around loss of power and control, overburdening the family, lack of health literacy and fears about being unable to establish a therapeutic alliance with patients due to family involvement [6, 7]. HCPs often underappreciate the unique skills and expertise that family members can provide both in their own family members’ care, as well as in the systems in which they have lived experience [3, 8]. Future directions for research highlight the need for an in-depth understanding of families’ views regarding this relationship [6, 9,10, 11].

The need for an in-depth understanding of the relationship between families and HCPs was also highlighted in a scoping review on relationship-centred care (RCC) [12]. RCC is a model that provides an alternative framework to patient-centered care by focusing on how all relationships influence healthcare experiences and outcomes. RCC emphasizes personhood, and values patients and HCPs as active participants who bring important aspects to the relationship. These interactions influence the course and outcomes of care [13,14]. The relational dimensions of RCC include: HCP–patient,–family,–self,–colleague,–community and–organization [12]. This review identified gaps in the literature within the relational dimensions of HCP–family.

We explored the relationship between family members and HCPs, specifically how family members can influence the course and outcome of patient care for youth. Exploring this under-researched area provided an opportunity to understand the tripartite relationship between the family, the youth experiencing mental health problems or substance use concerns and their HCP. Although we began the project by recruiting family members who were involved with caring for a youth with early psychosis, we quickly realized that the diagnosis was secondary to the experience of caring. Several caregivers who saw our recruitment poster contacted the study authors to describe how watching their youth descend into serious substance misuse was frightening and heartbreaking. Therefore, we opened recruitment to any family member who identified as being highly involved in caring for a youth receiving mental health and/or addictions services. We focused on youth (ages 16–29) because this is a time of transitions and challenges both in the shifts from child to adult services and a youth’s right to determine who will be given access to their health information, including family. Families can be allies to both the people they care for and the HCPs. Understanding how, in most situations, the family is essential to a youth’s health and well-being provides opportunity to redefine relationships in healthcare. Thus, the research question was: How do families experience the tripartite relationship between HCPs, themselves and youth patients with a psychiatric illness? We aimed to understand the type of relationships formed between HCPs and family members throughout a patient’s course of care, the family member’s perceived role in the care of their youth accessing mental health or addiction services and the family member’s awareness of formalized structures (i.e., hospital rules, policies) and resources that support family involvement.

## Methods

We used a qualitative research design and a constructivist grounded theory approach to data collection, and analysis was used to answer the research question [15]. In traditional grounded theory approaches, the researcher maintains the position of a “distant expert” who allows the data to emerge with as few pre-determined thoughts as possible. The interview data should not be influenced, filtered or interpreted by pre-existing biases or hypotheses of the researcher. In other words, grounded theory requires a “tabula rasa” or blank slate approach to data collection and analysis. We used a constructivist grounded theory approach because we believe that researchers are active participants in the research process. Our theoretical perspectives and the interaction between the interviewer and the interviewee during the interview process contributed to shaping the data collection and analysis.

This study was approved by the Centre for Addiction and Mental Health Review Ethics Board (Protocol #021–2017). We obtained verbal and written consent from all participants included in the study.

### Setting and recruitment

We used convenience sampling to identify potential participants [15]. Participants were recruited between May 2017 and February 2018 through advertisements placed strategically within the largest urban mental health and addictions hospital in Canada. At this hospital, there is a service specifically for transition-aged youth that offers outpatient services. As well, the Office of Family Engagement offers resources and navigational support for families. We did not specify or ask potential participants to disclose a diagnosis beyond being involved in caring for a youth with mental health and/or addictions issues. Recruitment focused on English-speaking participants over 18 years of age who considered themselves to be highly involved in the care of their young adult for at least one year. We chose this time period because it provided family members with an opportunity to reflect on multiple interactions and experiences with HCPs. We also did not want to overburden family members knowing that the first few months of a diagnosis can be an overwhelming and confusing time, making it difficult for them to participate and reflect on their experiences.

### Sample size

To identify the appropriate sample size, the five considerations associated with concept of “information power” were used: study aim, sample specificity, theoretical background, quality of dialogue and strategy for analysis [16]. Our study aim was narrow, we had a specific study population, we were using an established theoretical framework for data collection and for data interpretation, the interviews were semi-structured and conducted by one research analyst and our analysis strategy included an in-depth analysis of participants’ narratives. We met as a team to discuss our theoretical framework, the coding and the emerging themes throughout the data collection and analysis process. Data collection and analysis actually occurred simultaneously. When we noticed patterns in the data that were confirmed through repetition (approximately interview #18) it signaled to the team that there might be no new information discovered through more interviewing. However, we continued to conduct three more interviews to be reasonably assured that further data collection would yield no new results. Guided by these considerations, it was determined that 21 participants provided sufficient information power to capture the experiences of family members.

### Data collection

Given that family involvement in mental health services is highly complex and that common themes across international studies indicate that families feel isolated and unrecognized in care planning [17–20], we used semi-structured interviews to engage participants. This provided a balance between the flexibility of allowing participants to guide the conversation while remaining on topic. We had nine potential questions with various probes that we could ask the participants, with the aim to cover key factors. In other words, it was not necessary to complete the entire interview guide (S1 interview guide). Majority of the time, the participants answered questions that we had on the guide even before we were able to ask them. Most of the interviews lasted between 60 and 120 minutes.

All interviews were conducted in English, audio recorded and transcribed verbatim by a professional transcriptionist. The interviews were anonymized upon transcription and the audio files were destroyed upon verification of the data.

### Data analysis

A thematic analysis process was used, as described by Braun and Clarke [21], to review the transcribed interviews, generate codes, develop themes and present the findings in a final report. It is important to note that this was not a linear process; on the contrary, it was a recursive process, where the research team cycled through the phases until the satisfactory results at each phase were achieved. In **phase one**, all audio recorded interviews were transcribed verbatim and uploaded into NVivo 11. **Phase two** involved the production of initial codes from the data. The research team (SS, MM, AJ, JV) gathered to read through several transcripts to identify codes across the whole body of transcripts. These codes identified features of the data that were interesting and referred to a segment of the transcript that signified a concept related to participants’ experiences. In **phase three**, the research team arranged different codes into potential themes by collating all the relevant coded data extracts within the identified themes. **Phase four** involved two levels of reviewing and refining our themes. Level one involved verifying all codes within one theme to form a coherent pattern. Level two involved a similar process, but in relation to the entire data set. In this phase, we verified if our thematic map accurately reflected the meanings evident in the data set as a whole. In **phase five**, we conducted a detailed analysis for each theme. The detailed analysis entailed articulating the “story” that each theme told, and how that story fit into the overall conception of the data in relation to our research question. This step was necessary to ensure there was no thematic overlap between the themes developed in phase four. In **phase six**, we produced the final report on the findings and made connections between the story and the research question. Since we were interested in RCC, we used this framework to anchor our findings. This is in keeping with the constructivist approach to data analysis. Also in keeping with a constructivist grounded theory approach was the consensus building that the authors engaged in during the coding and data analysis phase. We acknowledge that our backgrounds as health professionals, education specialists, scientists and family members with lived experience interacted and shaped decisions around the naming of codes, their categorization, the thematic interpretation and the final conclusions for this research project.

## Results

Approximately 86% (n = 18) of participants identified as the mother of a youth; one participant identified as a father (mother and father interviewed together); one participant identified as a grandmother (interviewed with the mother) and; one participant identified as a sibling (brother). The sibling described being part of his sister’s care because his parents were not fluent in English and he was able to serve as a communicator among the parents and healthcare providers. Approximately 62% of participants (n = 13) described their youth as experiencing mental health issues; 29% of participants (n = 6) described their youth as experiencing both mental health and addiction issues; and 10% (n = 2) described their youth as experiencing addiction issues (Table 1).

**Table 1:** Participant information.

Participant ID	Gender (Male/Female/Other)	Relationship to youth	Participant reported issue
FV01	F	Parent (mother)	Mental health
FV02	M	Sibling (brother)	Mental health
FV03	F	Parent (mother)	Mental health and addictions
FV04	F	Parent (mother)	Mental health
FV05	F	Parent (mother)	Mental health
FV06	F	Parent (mother)	Mental health
FV07	F	Parent (mother)	Mental health
FV08	F	Grandmother	Mental health
FV09	F	Parent (mother)	Mental health
FV10	F	Parent (mother)	Mental health and addiction
FV11	F	Parent (mother)	Mental health and addiction
FV12	F	Parent (mother)	Addictions
FV13	F	Parent (mother)	Mental health and addiction
FV14	F	Parent (mother)	Mental health and addiction
FV15	F	Parent (mother)	Addictions
FV16	F	Parent (mother)	Mental health
FV17	M	Parent (father)	Mental health
FV18	F	Parent (mother)	Mental health and addiction
FV19	F	Parent (mother)	Mental health
FV20	F	Parent (mother)	Mental health
FV21	F	Parent (mother)	Mental health

All participants discussed the interpersonal process and relationship-building aspects of caring for their youth with a mental health problem or substance use challenge. Their responses were categorized into four relational themes with various sub-themes.

1. The family member–HCP relationship
  - Creating a positive impression
  - The extension of the patient
  - Pink flags
2. The family member–youth–HCP relationship
  - Receptivity of youth to familial involvement
  - A youth’s individual right to privacy while on a shared family journey
3. The family member’s relationship to self
  - A family illness
4. The family member’s relationship with friends, family and peers
  - Loneliness, stigma and shame
  - Lack of understanding about mental health problems and substance use

### The family–HCP relationship

Within the relational dimension of the family–HCP relationship, participants described three sub-themes about the importance of developing and maintaining a relationship between themselves and their youth’s HCPs to support treatment and recovery outcomes. First, they described the importance of creating a positive impression of themselves for HCPs. Second, they characterized themselves as an “extension of the patient” because they were deeply involved in the care of their youth; their experiential knowledge as a result of this involvement could provide valuable information to HCPs. Third, participants explained the concept of a “pink flag,” and described how their ability to recognize when their youth was starting to feel ill was significant and important to receiving timely treatment and signaling when recovery might be compromised. Below, we describe these sub-themes in more detail.

#### Creating a positive impression

Several participants described how they deliberately tried to make a favourable impression on their youth’s HCP to form a positive relationship. These participants viewed a positive relationship with HCPs as key to their youth’s medical treatment and recovery process. These participants shared how they presented an image of themselves as someone who was credible, knowledgeable, not too emotional, non-threatening and highly invested in the health of their youth. Once they gained the trust of HCPs, they were able to show vulnerability and engage in more authentic and meaningful conversations. One participant articulated:

I felt this desperate need to prove that I was credible.. .. So, my initial feeling was almost like a burdensome thing.. .. I had to prove my credibility, I couldn’t be emotional, I had to prove I was rational and objective and intelligent and had a good relationship [with SON]. I had to prove I’m someone you can listen to.. .. I could see them trying to size me up and figure out whether I was someone that was credible.. .. I was successful in doing that and people listen to me now, respect my opinion and don’t make me feel like I have to be the perfect mother all the time. [FV06]

Most participants believed that establishing rapport with HCPs encouraged the flow of information:

Once they got comfortable with me and they knew that I’m not going to abuse the power, then they lightened up and we have [sic] some good conversations. [FV14]

Other participants shared that some HCPs seemed intuitively good at allaying their concerns and fears while providing pertinent information from the very beginning, without the need for rapport building. This participant described how the younger generation of psychiatrists seemed to have mastered these skills:

I love the younger psychiatrists coming through. They’re so much better, right? It’s good to have the people that have the experience, but I find the new, the younger, psychiatrists are light years ahead of being able to interact properly with different types of people and family members as compared to the older ones. [FV04]

The quality of the family–HCP relationship was believed by several participants to play an important therapeutic role. Thus, participants wanted to make good impressions to develop trust, keep the lines of communication open and therefore better support their youth.

#### The extension of the patient

Participants described having intimate knowledge of their youth that reached far beyond what was shared with an HCP. Therefore, family should be considered an “extension of the patient.” The knowledge of families could be helpful when treatment decisions are made based on how youth are doing in their everyday lives:

[HCPs] certainly have the expertise on the condition that I don’t, but the piece that I do bring to the table is the expertise on the child. [FV05]

All participants agreed that including and valuing their experiential knowledge as an extension of the patient will positively impact their youth’s care. This would be particularly important in circumstances when youth might downplay, be uncommunicative or outright deceptive about what is happening in their everyday lives:

She’s a smart kid and she would choose selective information to share with the healthcare provider, whether the psychiatrist or whoever. And it was frustrating for us as parents because there was another side of the story that wasn’t being heard by the psychiatrist. [FV19]

Family members also found their relationships with HCPs were strengthened when they saw what they perceived as excellent care for their youth:

I think the experience of just being with [HCPs] and seeing the way in which they are supporting my daughter, hearing some of the really good questions that they’re asking her has helped. Watching how they’ve progressed through just looking at my daughter’s needs.. . She asked really good questions, and I guess earned my respect. [FV07]

Given these beliefs and experiences, participants shared a common frustration when they perceived their contribution to care was misunderstood or dismissed by HCPs:

I'm not here to provide the care, you are, but I am his rock. I am his support. I know how he thinks. I know what home life is like. I am that piece of the puzzle and you need to consider me an equal. I'm that piece of the puzzle that is going to be vital for you looking after a mental, emotional dysregulation, everything that I am such a huge part of. I am such a vital part of his life, and always will be, and you want to shut the door on me? It just doesn’t make sense. [FV05]

#### Pink flags

Many participants described pink flags as instances when their youth’s behaviour was “slightly off,” but not so out of character that immediate help was required. These instances were also called “gut feelings,” and were considered to be early warning signs about their youth’s behaviour. One of the participants explained her experience of a pink flag:

Driving home that night, DAUGHTER called me and said that she relapsed. It’s funny, too, because earlier at that meeting, I had said to THERAPIST, “Things are good, but you know, I sense something.” She goes, “Oh, you have got a pink flag on your shoulder, not a red flag, a pink flag.” I said, “Yes.” [FV13]

Almost all of the participants explained that they noticed things that were “not right,” or out of character, far earlier than HCPs could. They learned to “listen to their gut” because it always proved to be right:

This sounds very unscientific, but one thing this whole experience has taught me is to trust my gut. I think the gut of a supportive, healthy family is very meaningful. Sometimes, I’ll just say, things don’t feel right. And, it will be true. Things aren’t right and it’s the beginning of something going wrong. [FV06]

Given the challenges of describing a gut instinct or of conveying signs of worrisome behaviours, some participants described the difficulty of taking action when they noticed a pink flag:

I should have said I’m not taking her home. I knew there’s something wrong with her but again, [HCPs] are the voice of authority. I don’t want to say I’m just the mother but I’m the mother. Again, I’m not the PhD, what do I know? These people deal with this every day.. .. I take her home like an idiot. By the next day my daughter was out of it. The long and short of it, I got my daughter to the hospital, thank God. [FV 19]

Overall, the majority of participants believed they had early and important knowledge of their youth’s health, which could help facilitate their youth’s care and recovery outcomes.

### The family member–youth–HCP relationship

Families described two sub-themes within this important tripartite relationship that influenced care outcomes. The first was the receptivity of their youth to family involvement and to family communication with HCPs. Sometimes receptivity was automatic, and other times it was negotiated. In either case, this receptivity had a perceived impact on care and recovery outcomes. The second sub-theme was their youth’s individual right to privacy on what participants described as a “family journey.” Although all participants discussed the need to respect their youth’s privacy, there were instances when privacy and confidentiality were perceived as secondary to issues of safety and recovery outcomes.

#### Receptivity of youth to familial involvement

Some participants described feeling very fortunate that the relationship they had with their youth prior to the onset of their illness seemed to facilitate their involvement in care:

There’s been good dialogue and in fact, we’ve been lucky that DAUGHTER hasn’t shut us out, hasn’t said, “No, you’re not able to come to the appointments.” Because that would be hell. [FV01]

In fact, for some participants, a lack of receptivity to familial involvement would signal that their youth might be more ill, or that there may be other unaddressed issues:

That would mean that she was a lot iller [sic], you know what I mean? I think she’s well enough to know that it’s a good thing that we’re working together to be part of it. For her to shut us out would mean, to me, that there were more issues and it would be harder. [FV03]

Other participants described their own “withdrawal” from care as a natural progression and an indicator of their youth’s growing maturity and increasing wellness.

Several participants described that it was difficult when their youth did not want them involved in their care. They described this experience as an “in-between” because biologically, their youth were considered adults, but socially and cognitively, they were often unable to make “adult” decisions. As family members caring for their youth, there was a natural pull toward trying to protect them from certain choices, but also an understanding that “shielding them” might actually be harmful in the long run:

I always say, these kids are making adult choices and they’re children. You have to allow them to take responsibility and feel the consequences of their adult choices. And, oftentimes, we shield them. We shield them from going to jail, we shield them from this, we shield them from that, thinking that we’re protecting them, but we’re not, we’re helping them live in a magical world. [FV09]

This “in-between” stage was also described by participants as a time when their youth felt a natural desire to exert their independence:

Part of the problem is, sometimes, my son doesn’t want me involved. Which, I think probably happens quite a bit. Although we have a really great relationship and he talks to me and everything, but it’s his desire for independence and not always understanding. [FV14]

A few of participants also believed that youth might not want family involvement because they could “manipulate” their HCP more easily:

And I also think that a lot of young people, I can speak for our son, can be a little bit manipulative and say that things are rosier than they actually are. And great, then I won’t have to keep coming to these stupid Wednesday night meetings with my psychiatrist by saying everything’s hunky-dory, and it’s not. I think despite being skilled in their craft, I think the psychiatrist could miss some of what’s happening at home, if there was no family contact. [FV10]

Overall, participants emphasized the importance of communication with HCPs as a way for families to guard against their youth’s deception or downplay of what is actually happening.

#### A youth’s individual right to privacy while on a shared family journey

The majority of participants believed that because of the ongoing nature of family involvement in care, the journey into the mental healthcare system was not an individual journey but a family journey. Thus, many participants were surprised to learn that without their youth’s consent, they would not be able to communicate to HCPs about treatment and care:

And when I went to see her psychiatrist at HOSPITAL with her, they were telling me that she has consent rights at 12 years of age. And I was shocked. I was completely shocked and insulted. Are you telling me that my daughter has to consent to medication, has to consent to record release? I can be removed from here altogether? And she has consent to a whole bunch of privacies.. . I was like in shock. I was floored. [FV16]

As discussed, all participants felt that they had a unique and important perspective that would help HCPs effectively care for their youth, and so the potential of having this perspective “blocked” was often met with shock and dismay. The most challenging aspect of being unable to communicate with HCPs came when participants perceived their youth to be “too ill” to make “good” decisions. In these cases, some participants believed that the right to safety was more important that the right to privacy:

Now, there are times where a person’s well-being and safety trumps their right to privacy. Well-being and safety are rights too. Far bigger rights, really, than privacy. And, there comes a time where you’ve got to look at, okay, which right is more important at this point in time? [FV17]

Some participants felt that the primacy of maintaining privacy when their youth was ill was detrimental to their overall recovery:

In my view, the Privacy Act is ridiculous in those circumstances when there’s concurrent disorder, when there are kids using drugs that are obviously damaging, harmful to their brains. And they’re using to the degree where they stop going to school.. .. She was sick, and for me not to be involved, for parents, for family not to be involved because of a Privacy Act, I was like, how can that be? To me, it’s over the top. Same with the consent to treatment, that’s totally over the top. [FV 11]

Several participants believed that when their youth was in crisis, consent to treatment did not always make sense because their youth was not in a position to make good decisions about treatment options:

And, he needed to have choices taken away from him. That’s the thing. You’re always allowed to have choices here. When kids are on drugs, they don’t make good choices.. .. There’s no motivation for them to make a good choice, so why pretend that they should be allowed to make their choices? That was really frustrating, that so much was up to them in their own treatment. [FV09]

Other participants discussed strategies that they used to help bridge the communication gap:

I couldn’t get information from them [HCPs], but they freely accepted information from me, and I think that was a good way of handling it to get around the confidentiality because I provided them with open access to what was happening in my life. And, thereby, they were able to use that to patch together a bit fuller picture of what was going on with SON. [FV18]

However, this participant described how difficult “not knowing” was as a caregiver:

During his first admission, he wouldn’t allow me to visit, and nobody [sic] to talk to me or anything. He forbade them [HCPs to talk to me]. So, that was probably the most difficult time I’ve ever had. That was brutal. But, they were not allowed to share, not even allowed to respond to my e-mails. When he finally opened up and let me talk to them, I got e-mails from people apologizing, saying, “I’m sorry, we were reading your e-mails, but we weren’t allowed to share, because he had asked us not to.” .. . He didn’t even want them to share that they’re not allowed to share with me.. .. It was actually cruel. It was cruel for a mother to have their kid in a situation like that and not have any information. [FV06]

According to this participant, HCPs walk a “fine line,” and a thoughtful explanation of this delicate balance would help family members understand their potential role within the boundaries of privacy and confidentiality.

### Family member’s relationship to self

Within this relational dimension, one main sub-theme emerged from the interview data: Self-care becomes embedded within the context of mental illness and addiction as a family illness. Participants described how their time, energy and mental and physical health were personally affected as a caregiver.

#### A family illness

The majority of participants described the way the whole family is affected by an illness when one person has mental health problem or substance use challenge:

The family needs to learn to take care of themselves too, because we are just getting as sick as them. It’s a family disease and you can’t take care of one and not take care of the other. [FV13]

They also discussed the emotional and physical toll of caring for their youth, which left little or no time for self-care. Many participants described the family experiencing “collateral damage” because they developed their own health issues:

There was a lot of collateral damage in the family from SON's illness. I developed a lot of health problems from living in that high-level stress.. .. We never left the house. We always had to be home to watch him. Since he got ill, I developed a stomach condition just from stress, which is total inflammation of the stomach lining and it went on for years. .. and then my daughter developed anxiety issues, which she still has. He’s doing better now, but my daughter still has problems, and I don’t know what to do about that.. .. I think the mental health of the family needs to be considered a bit more.. .. It’s not like being a care provider who gets to go home at the end of the day. It’s like being in combat, so I think it’s really important that the lifestyle of the family be considered in part of that patient care. [FV18]

Some families described the therapeutic distance they created between their youth and themselves, particularly when certain behaviours were difficult to understand or accept:

I’ll be honest with you. There have been times where I have therapeutically distanced myself physically for my own safety, but it’s still been framed in “I love you very much, right now I don’t like what you’re doing, this is not healthy, it is not safe, and here are the consequences, and let’s work with that.” So, even in those instances, the communication to her has been delivered in a way in which it is a tool for her to develop more motivation and a little more insight into her behaviour. [FV12]

However, several participants recognized that the balance between involvement and distance is difficult to determine:

And it’s tough because it’s really hard to know what to do, how much to be there. .. and how much to stand back. [FV01]

Although the participants described the emotional and physical toll of caregiving, they emphasized how they remained highly motivated to be supportive and involved in the care of their youth.

### Family member’s relationship with friends, family and peers

Within this relational dimension, two sub-themes were identified about the importance of peer support. First, participants described the loneliness that results from feelings of stigma and shame about mental illness and substance use. Second, they explained how a lack of understanding about mental health problems and substance use impacted their relationships, and thus their ability to get support from friends and family.

#### Loneliness, stigma and shame

The majority of participants described experiencing loneliness and isolation, and a lack of understanding from extended family members, friends and society in general. They attribute this isolation to the stigma and misconceptions around mental health problems and addictions:

It’s not like he had cancer.. .. If he had cancer, everybody would have rallied around, but not with mental illness. It’s a choice to keep it a secret anyway because of social stigma, because. .. people have an idea from television and movies about who gets mental illness. There is always horrible child abuse in the family, so they are looking at you differently. Only bad people or people from bad families get [mental illness]. I honestly would have felt the same way had it not happened to us because that was all the information I had ever gotten in my life about schizophrenia; something was done to people so that they became [mentally] ill. [FV20]

Several participants found that friends and family who had no experience with mental illness or addiction could not adequately support or fully understand their situation. In these cases, support groups were essential supports for families:

The reason why we liked going [to support groups] so much is you can’t talk about that stuff to just your best friend who’s never gone through it. Like, they just look at you.. .. I had some people that I would share my story with, you know, some of my best friends, and they’d stand there and just ball their eyes out because of my journey. I was like, I don’t know if this is good for me. So going to that peer setting, I got so much out of it because you could relate to each other’s story. [FV11]

The ability to access peer support helped to alleviate the need for “keeping secrets” and reduced the feelings of shame that often surround mental health problems and substance use:

You feel like you’re kind of lost on an island. [Peer support] certainly reduces your shame. You know that there are many others that are facing the same issues as you are. And to get to verbalize and vocalize it with somebody who actually lives it and understands it is so shame reducing. [FV10]

The need for peer support was seen as essential, given the loneliness, isolation and shame that families experienced as a result of caring for their youth with mental health problems and/or substance use challenges.

#### Lack of understanding about mental health problems and substance use

All participants felt that there was a general lack of understanding about mental illness, which impacted their relationships within healthcare institutions and their ability to receive empathetic care:

My first child had complex medical needs. He had a respiratory and a cardiac condition. In order to feed him, I had to de-suction him. De-suctioning caused irregularity in his breathing, so I had to do chest physio. And once the suctioning and chest physio were over, I would feed him.. .. Without a shadow of a doubt, it was easier to take care of the kid that couldn’t eat or breathe than it was to take care of the kid with mental health challenges. The system was empathetic. The system never questioned me or very rarely questioned me. They would give support. He had access to multiple clinics at HOSPITAL. They understood the stresses of dealing with a child like that. And there was, frankly, no comparison. [FV12]

A few participants revealed that they had no concept of mental illness and addiction prior to caring for their youth. They joined peer support groups to increase their own understanding of their youth’s illness:

I joined a support group specifically to understand the disease more, because I didn’t want to just hear it from people that were care providers. I needed to understand what was going on in his head and I figured that people who were experiencing it were the best source of that information. [FV15]

Participants described how their relationships with others with similar experiences helped them better support their youth and themselves while navigating the mental healthcare system.

## Discussion

### Main results

Family members experienced and conceptualized their relationships with HCPs, their youth, themselves and their friends and peers as active interactions that influenced the course and outcomes of care. Similar to other research findings, we found that families recognized the importance of privacy and confidentiality [22]. In fact, several participants reiterated that they did not need to know everything about the youth they were supporting. However, because family members were often able to identify pink flags, they felt the need for a greater role in the care and recovery of their youth. There is an extensive literature on the role of physicians’ “gut feelings” or otherwise referred to as intuitive medicine [23]. A systematic review describes this “sense of alarm”, “gut feeling” or intuitive medicine as an important diagnostic tool for physicians [24]. It will be important to consider the impact of the family’s role in raising “pink flags” and its contribution to the “gut feelings” of psychiatrists in the diagnosing and treatment of youth with mental health and substance misuse.

What was novel in this work was participants’ descriptions of their need for “impression management” to gain credibility and open up the lines of communication between themselves and their youth’s HCPs. Impression management is an attempt to influence and improve a person’s image in the eyes of others. Goffman coined the phrase and further described how we create impressions through “sign vehicles” that include both verbal and non-verbal communication [25]. Several participants used the “manner of interacting” sign vehicle as a way to get HCPs to form favourable impressions of them, and thus deem them credible and trustworthy of giving and receiving information. Many participants described the effort they made to create this impression, which served as their gateway to becoming an extension of the patient and having their pink flags taken more seriously by HCPs.

All participants felt that they had important information that would help HCPs more effectively care for their youth. Although all participants demonstrated respect for their youth’s confidentiality and privacy, the potential of having this perspective “blocked” by an overcautious HCP was considered to be a barrier to providing support and care for their youth. One participant described how she knew more about the care of her child who has severe physical disabilities than she did about the care of her child with a mental illness. Researchers have found that these barriers contribute to less effective treatment outcomes that result in higher administrative and resource costs [26]. Several participants suggested that organizations provide clear and specific information on what families can expect and the various ways information can be exchanged to improve the care of their youth. As one participant noted, they did not know that their phone calls and e-mails were being listened to, read and considered until weeks after their youth consented to having the family notified.

Participants described how a stigma still exists around mental health and substance misuse in society. Research shows that people associated with individuals with mental illness or addiction can be stigmatized because they are in some way connected to someone with a “stigmatized identity” [27]. Some participants also described feeling socially isolated because of the myth that “bad parenting” or “bad families” are the cause of mental illness. This myth is perpetuated through the news, movies and other forms of media that represent mental illness and substance use in shocking, violent or otherwise sensational ways which do not necessarily represent most people’s experiences. Television shows and movies tend to depict mental health and addictions in ways that reinforce popular culture rather than scientific understandings [28]. For example, there is an over-representation of negative portrayals of people with mental illness or substance use as violent or dangerous to others in the media [29, 30, 31]. Thus, the stigma of mental illness and its associated myths and misconceptions prevented some participants from sharing their experiences with friends and family. This was described by some participants as a very isolating experience. Peer support was deemed essential for the majority of participants. Only one participant described feeling too exhausted to support others and thus avoided peer support. Overall, the majority of participants in the study felt that the family peer support they received was essential in reducing their isolation and feelings of shame. They described feeling validated knowing that they were not alone and that there were other families that understood their challenges. Given the importance of peer support, it would be important for organizations to offer a wide range of support options that could meet a range of family needs.

### Strengths and limitations

We were provided rich, in-depth information about the importance of family involvement in the care and health outcomes of youth who are accessing mental health and addiction services. Although findings in qualitative research are not generalizable, our findings are transferable and support the findings of other research studies. We only interviewed family members and so their responses are based on their subjective interpretation of events that may not reflect the multiple perspectives of others who are involved in care. Interviewing family members who describe themselves as not being involved in the care of their youth could also yield important considerations. Triangulating perspectives by interviewing HCPs and youth patients could have provided a more detailed perspective of this complex issue. We focused solely on the family perspective because it was lacking in the academic literature; however, future studies are needed to collect these multiple perspectives.

## Conclusion

Participants were invested in their youth’s care and made efforts to be relatable and gain legitimacy in the eyes of HCPs, a strategy for facilitating trusting and therapeutic relationships. They used their experiential knowledge and identification of pink flags to contribute to their youth’s treatment and recovery. Many participants want HCPs to consider them as an extension of the patient to change the culture of fear surrounding privacy, which can prevent information sharing in an effective manner. Participants believed that mental illness was a family illness, and thus a family journey toward recovery and better health outcomes. A family-centred approach that focuses on peer support for families, education and transparency about privacy and relationship-building among families, youth and HCPs is necessary to overcome the existing barriers to the inclusion of families in the care of their youth.

## Supporting information

S1 Interview Guide (DOCX)
