# Supplementary material for: Where is the family voice? Examining the relational dimensions of the family- healthcare professional and its perceived impact on patient care outcomes in mental health and addictions
Source: PLoS One. 2019 Apr 12;14(4):e0215071. doi: 10.1371/journal.pone.0215071 (PMC6461270; doi:10.1371/journal.pone.0215071)
Supplement: S1 Interview Guide — (DOCX) [file pone.0215071.s001.docx]

**Appendix A: Semi-Structured Interview Guide**

1. How are you involved with helping your family member maintain their health (day-to-day care, during times of hospitalizations or medical appointments)?
   1. How would describe your relationship with your family member when it comes to caring for them?
   2. How would you describe your relationship with your family member’s care provider(s) when it comes to caring for them?
2. How do you find out about what is going on with the medical care of your family member?
   1. What kind of information is provided? Is it enough? Is it easy for you to find out about things that would help you care better for your family member?
3. Do healthcare providers promote family involvement? Can you give examples of when family involvement is welcome and when it is not?
4. How do you communicate with healthcare providers about your family member? Are your expectations, needs and perspectives taken into consideration by healthcare providers in relation to the care you provide for your family member?
   1. Probe: If there is no communication, how would you like to communicate?
5. How important is it for families and healthcare providers to have a relationship with one another for the care of a loved one?
6. What communication challenges do families face when they try to become involved in the care of their loved ones?
7. From the healthcare provider perspective, the literature suggests that there are some concerns/barriers to involving families during treatment:
   1. privacy concerns;
   2. patient fears of placing relatives in a position of power or of exposing their vulnerability,
   3. it may interfere with the physician-patient relationship,
   4. Fear of burdening the family and worsening the patient’s symptoms.
      1. Probe: What are your thoughts and experiences regarding these concerns?
8. Are there times when it would be inappropriate to involve families in the care of a loved one?
9. Do you know if the hospital has any policies that promote family involvement in patient care? Would a hospital policy help? What are some of the key elements that it should contain to be supportive of family involvement?

*General probes: Can you explain more about that/; What do you mean when you say X?
